# Sleep in People with Dementia and Their Caregivers

**DOI:** 10.1002/alz.091005

**Published:** 2025-01-03

**Authors:** Carol A Manning, Anna E Youngkin, Mark S Quigg

**Affiliations:** ^1^ University of Virginia, Charlottesville, VA USA

## Abstract

**Background:**

There is mounting evidence that difficulties with sleep including insomnia, sleep quality, and sleep fragmentation contribute to Alzheimer’s disease risk including formation of beta‐amyloid. Disrupted sleep is common in people with dementia (PWD). Primary unpaid caregivers (CGs) of PWD may also have disrupted sleep as a result of their caregiving roles. It is clear that CGs experience CG burden, stress and depression. The interplay of CG‐PWD dyads and the impact of behavior, cognition and sleep in the PWD on the CG are relatively understudied.

**Method:**

Eight dyads consisting of individuals diagnosed with Alzheimer’s Disease (AD) (PWD) and their CGs were recruited from the Virginia Alzheimer’s Disease Center (VADC) Clinical Cohort. Diagnosis of AD in the VADC cohort was made by team consensus using neuroimaging, biomarker, neurological and neuropsychological data. All participants had wrist actigraphy measured for 14 days and overnight polysomnography (PSG) done in a sleep lab. Dyad members were in separate rooms during PSG.

Dyads members independently completed the Montreal Cognitive Assessment 12, the Geriatric Depression Scale 13, Epworth Sleepiness Scale 14, and the Quality of Life in Alzheimer’s Disease scale 15 (QOL). CGs only completed the Preparedness for Caregiving Scale 16 and the Zarit Burden Interview and the Neuropsychiatric inventory.

**Result:**

Neither activity nor rest over 14 days differed within dyads. Relative to PWD, CGs experienced shorter total sleep, lower sleep efficiency, and longer sleep onset. CG and PWD depression and QOL did not differ between dyad members. Importantly, poorer CG sleep was associated with higher CG burden and greater PWD neuropsychiatric symptoms but not with degree of cognitive impairment in the PWD.

**Conclusion:**

Results suggest that sleep in CGs can be more impaired than the PWDs they are caring for even when sleeping separately. Importantly, behavioral disturbance may be more problematic than PWD cognitive impairment level in CG sleep. These may have implications for ability to care for the PWD potentially impacting nursing home placement. Furthermore, PWD sleep characteristics may impact longer‐term CG health and well‐being.

